# Short-term effects of amelogenin gene splice products A+4 and A-4 implanted in the exposed rat molar pulp

**DOI:** 10.1186/1746-160X-3-40

**Published:** 2007-12-21

**Authors:** Nadège Jegat, Dominique Septier, Arthur Veis, Anne Poliard, Michel Goldberg

**Affiliations:** 1 Oral Biology, EA 2496, Faculté de Chirurgie Dentaire, Université Paris Descartes, Montrouge, France; 2 Feinberg School of Medicine, Northwestern University, Chicago, USA; 3 Laboratoire de Différenciation, Cellules Souches et Prions, CNRS-FRE2937, Villejuif Cedex, France

## Abstract

In order to study the short-time effects of two bioactive low-molecular amelogenins A+4 and A-4, half-moon cavities were prepared in the mesial aspect of the first maxillary molars, and after pulp exposure, agarose beads alone (controls) or beads soaked in A+4 or A-4 (experimental) were implanted into the pulp. After 1, 3 or 7 days, the rats were killed and the teeth studied by immunohistochemistry. Cell proliferation was studied by PCNA labeling, positive at 3 days, but decreasing at day 7 for A+4, whilst constantly high between 3 and 7 days for A-4. The differentiation toward the osteo/odontoblast lineage shown by RP59 labeling was more apparent for A-4 compared with A+4. Osteopontin-positive cells were alike at days 3 and 7 for A-4. In contrast, for A+4, the weak labeling detected at day 3 became stronger at day 7. Dentin sialoprotein (DSP), an *in vivo *odontoblast marker, was not detectable until day 7 where a few cells became DSP positive after A-4 stimulation, but not for A+4. These results suggest that A +/- 4 promote the proliferation of some pulp cells. Some of them further differentiate into osteoblast-like progenitors, the effects being more precocious for A-4 (day 3) compared with A+4 (day 7). The present data suggest that A +/- 4 promote early recruitment of osteogenic progenitors, and evidence functional differences between A+4 and A-4.

## Introduction

For more than 75 years dental surgeons have used calcium hydroxide for pulp capping [15] after an accidental pulp exposure during the removal of carious dentin or the preparation of a cavity. The sequence of events leading to reparative dentin formation is well documented, but some cellular and molecular mechanisms still need to be clarified. As a result of the high alkaline pH of Ca(OH)_2 _a scar is formed at the surface of the exposed pulp. Eventually, some pulp cells are committed, proliferate and differentiate into odontoblast-like or osteoblast-like cells producing an extracellular matrix (ECM). This structure can mineralize and form a reparative dentinal bridge [31] but the dentinal bridge may be inhomogeneous and display fissures where pulp remnants are located, forming a defective barrier, unable to prevent the pulp from bacterial contamination. Nevertheless, for decades this was the only biological approach aiming to heal and keep the pulp alive.

[21,22] and [29,30] were pioneers in exploring the therapeutic effects of bioactive molecules, namely Bone Morphogenetic Proteins (BMPs). BMPs are implicated in the early development of the tooth [14], and are also present in the mature dentin [9]. In addition, BMPs and their receptors have been identified in the dental pulp [35,36]. Pulp capping with BMPs stimulate the formation of reparative osteodentin. Since that time, other dentin ECM molecules were also shown to enhance reparative dentin formation (see for reviews: [12,13]).

In dentin and pulp, collagens constitute the major components, however collagen fibrils function more as potential carriers rather than bioactive molecules. Therefore an increasing interest has been directed toward a series of dentin collagen-interactive non-collagenous proteins (NCP). NCP molecules include firstly phosphorylated proteins, among which the SIBLING family appears to play major roles, especially as mineralization modulators [10]. Dentin sialoprotein (DSP), dentin glycoprotein (DGP), and dentin phosphoprotein (DPP) result from the cleavage of the native dentin sialophosphoprotein (DSPP). DSPP is expressed in a contiguous region on the long arm of chromosome 4q 21.3 5 together with dentin matrix protein-1, osteopontin (OPN), bone sialoprotein (BSP), and MEPE. Amelogenins and decorin are also phosphorylated, but to a lesser extent. Phospholipids associated with proteins as proteolipids, have also been identified as dentin ECM molecules.

Secondly, non-phosphorylated proteins [osteocalcin, osteonectin, small leucine-rich CS/DS and KS proteoglycans, serum-derived glycoproteins, enzymes such as alkaline or acid phosphatases, metalloproteases, disintegrins, and a limited number of growth factors (TGFβ, ILGF-1 and -2, FGFs)] belong to the family of NCP dentin proteins.

Phosphorylated and non-phosphorylated ECM components may function as structural proteins, as matricellular molecules with the capacity to link to the cell cytoskeleton, or as biological mediators of cell functions. In the later capacity they may 1) function as signaling molecules, 2) regulate growth factor, cytokine or hormone production, and 3) control the availability or activity of proteins sequestered within the ECM, binding or releasing growth factors (GF) and hormones [3,1]. As multifunctional proteins, ECM molecules have the capacity to be involved simultaneously in each of the three facets. Altogether, they are prominently involved in the formation, structure and mineralization of dentin and bone. In addition, some of them may promote pulp healing.

We have previously investigated the bioactivity of two amelogenin gene splice products A+4 and A-4, which induce either the formation of a dentinal bridge closing the pulp exposure (A+4), or promoting the massive formation of reparative dentin, both in the crown and root pulp (A-4) [33,19]. To summarize what is actually known firstly, a low molecular weight (6 – 10 kDa) polypeptide isolated from the rat incisor dentin matrix was found to have the capacity to stimulate *in vitro *embryonic muscle fibroblasts to express sulfated proteoglycans and type II collagen. For this reason the molecule was designated originally as being a Chondrogenic Inducing Agent (CIA) [2]. Secondly, the CIA was identified as a low molecular mass amelogenin polypeptide [23]. Thirdly, Veis and his collaborators [40] identified in the rat incisor tooth odontoblast/pulp library two specific cDNAs, the first resulting from expression of amelogenin gene exons 2, 3, 4, 5, 6 d, and 7 [A+4, 8.1 kDa] and the second from exons 2, 3, 5, 6 d and 7, the expression of exon 4 being omitted [A-4, 6.9 kDa]. Added to the culture medium, A+4 stimulated Sox9 expression whereas A-4 stimulated Cbfa1 mRNA expression. In the mouse enamel organ, the larger full length (M194) and near full length (M180) amelogenin isoforms are the principal products directing the formation of the mineralized enamel. The M194 mRNA includes both the full exon 6 and exon 4 sequences, while M180, the major mature amelogenin mRNA, still excludes exon 4. The corresponding short forms, M73 (A+4) and M59 (A-4, LRAP) include, or exclude the 14 amino acid sequence encoded by exon 4.

M194, M180 and M59 proteins are all produced, at different levels, within the mouse enamel organ, with M73 being present at much smaller levels. However, M73 and M59 are expressed by odontoblasts at the perinatal development period [37,16]. It has been postulated that the odontoblast produced M73 and M59 are secreted into the ECM, and during the period before dentin mineralization blocks ameloblast-odontoblast communication, they are taken up by the cells, where they perform their signaling function. Internalization of M59 (A-4) appears to be mediated by the cell transmembrane receptor LAMP-1, originally identified as a lysosomal membrane protein, in ameloblasts, odontoblasts and stratum intermedium cells [38,32]. Altogether, *in vitro *and *in vivo *experiments suggest that these amelogenin gene splice products are signaling molecules [39,19]. It was on this basis that the M59 and M73 were studied for their potential in dentin repair.

In *in vivo *implantation studies carried out for periods of 8 d, 15 d, 30 d and 90 d we observed the formation of reparative dentin or diffuse mineralization 2 weeks after implantation of A +/- 4 in the exposed pulp of rat maxillary molars [33]. In parallel, to get a better understanding of the mechanisms involved, the *in vitro *effects of A+4 and A-4 on clones of odontoblast progenitors were analyzed [26]. When A+4 or A-4 were added to the culture medium, RT-PCR analysis showed that it takes 48 h to stimulate the expression of DSP and osteocalcin [19]. From *in vitro *and *in vivo *studies it may be concluded that in the cascade of events leading to cell commitment, proliferation of progenitors is initiated between 24 to 48 h after exposure to A +/- 4, while the terminal differentiation occurs about 7 days after implantation. Consequently, we decide to investigate *in vivo *the short-term effects of A +/- 4 implantation.

## Materials and methods

100 μg of A+4 or A-4 were dissolved in a 150 ml solution of PBS. Affi-gel agarose microbeads (70–150 μm in diameter, Biorad, Hercules, CA) were incubated in the A+4/PBS or A-4/PBS solution for 1 hour at 37°C. Each bead absorbed approximately 0.2 μg of A+4 or A-4.

### Operative procedure

The preparation of the animals and pulp exposure was carried out as previously reported [33]. In brief, after gingival electrosurgery, half-moon-shaped Class V cavities were prepared on the mesial aspect of the first maxillary molars. Pulp perforation was accomplished by pressure with the tip of a steel probe. Soaked (A +/- 4 groups) and unsoaked (control group) agarose beads were implanted into the pulp, and the cavity was filled with a glass ionomer cement (Fuji II, GC Corporation, Tokyo, Japan) to prevent bacterial contamination.

### Bead implantation

Ninety maxillary first molars from 45 Sprague-Dawley rats, aged 8 weeks, were used (~350 g). The experiment was carried out according to regulations for animal care and approved by the Scientific Committee of the Dental Faculty.

The two **experimental **groups of 36 teeth (group A-4) and 32 teeth (group A+4) were implanted with small molecular weight amelogenin-soaked agarose beads. Four to six beads were implanted per pulp. Both groups were distributed into three subgroups to be evaluated at 1, 3 and 7 days respectively.

The **control **group included 22 teeth implanted with agarose beads alone. This group included 4 teeth at day 1, 12 teeth at day 3 and 6 teeth at day 7. We have previously reported the effects of sham preparations, calcium hydroxide and implantation of other bioactive molecules, using these same methods, the same strain of rats and therefore these experiments were not repeated [7].

### Specimen preparation for light microscopy evaluation

After 1, 3 and 7 days, respectively, the rats were killed by cardiac perfusion with a fixative solution containing 4% paraformaldehyde buffered with 0.1 M sodium cacodylate at pH 7.2–7.4. Block sections including the three molars were dissected from the maxilla, immersed in the fixative solution for 24 h at 4°C, and demineralized with 4.13% EDTA pH 7.2–7.4, renewed each 3 days for 8 weeks. The dehydrated tissues were embedded in Paraplast (Oxford Labware, St Louis, MO, USA). Five μm thick serial sections were cut, dewaxed and stained with hematoxylin-eosin or Masson's trichrome to evaluate tissue responses.

### Immunohistochemical evaluation

Proliferation was evaluated by immunodetection of the proliferating cell nuclear antigen (PCNA) [6,28], using a mouse monoclonal PCNA antibody (PC-10, Dako, Golstrup, Denmark) at 1/75e dilution in PBS-1% BSA.

Adjacent sections were labeled with the following primary antibodies: rabbit anti-RP59, a marker for osteoblast progenitors implicated in osteoblast recruitment [41] (kindly provided by Dr T. Wurtz, Paris 7) at 1/500 dilution, with anti-osteopontin (OPN) (LF 153) and anti-dentin sialoprotein (DSP) (LF153) at 1/100 dilution (kindly provided by Dr L Fisher, NIDCR-NIH, Bethesda, Maryland, USA).

The sections were further incubated with the secondary antibody with 1/100 dilution of peroxidase-conjugated goat anti-mouse IgG for the PCNA visualization or with a peroxidase-conjugated swine anti-rabbit IgG at 1/100 concentration for RP59, OPN and DSP (Dako, Golstrup, Denmark). The antibody localization was visualized with a solution of 3–3' diamino-benzidine (DAB, Sigma, St Louis, MO, USA) and H_2_O_2 _for 20 minutes. Controls were carried out by omitting the primary antisera from the labeling procedure, and using the secondary antibody alone. Previous immunoblot studies have assessed the presence of the molecules in the tissue and the specificity of the antibodies.

## Results

### Markers

The experiments were designed to discriminate between the *in vivo *effects due to the surgery and implantation of the untreated beads and the cascade of events resulting from the implantation of beads containing the amelogenin gene splice products into the pulp. Four markers were used to characterize the events occurring during the first 7 days after pulp implantation. The first marker was aimed at assessing the stimulation of cell proliferation. The antibody raised against the proliferative cell nuclear antigen (PCNA) was selected because it provides a reliable immunocytochemical method for visualizing the dividing cells within a tissue [6,28]. The other three markers, RP59, OPN and DSP, are less reliable in the sense that they may be less specific. Thus, it is the combinations of these markers that must be compared in order to discern their differential activity.

RP59, is a protein expressed in bone marrow cells and osteoblasts with implication in osteoblast recruitment, and it has also been detected in primitive mesenchymal cells, erythroid cells, and megacaryocytes [41,17]. RP59 is also a marker of differentiating odontoblast progenitors [19]. However, RP59 has also been identified in the forming enamel and ameloblasts [18], and therefore the specificity of this marker is questionable. Osteopontin (OPN) is a matricellular molecule and a structural protein prominently present in mineralized tissues [34]. As a member of the SIBLING family [10] it is phosphorylated to different extents in the ECM in different tissues. *In vitro*, OPN is a mineralization inhibitor [4,25]. It also functions as a response to stress and a mediator of inflammation [11,8]. Thus OPN is not an exclusive marker of the osteoblastic phenotype. Similarly, for years, DSP a peptide derived from the SIBLING protein DSPP by specific proteolytic cleavage was believed to be odontoblast-specific. However, its presence has also been recognized in osteoblasts in a 1/400 ratio [27] and in a few non-mineralizing tissues such as kidney, salivary glands and tumor cells [25]. Although the specificity of each marker remains a matter of discussion, at least the three of them altogether allow characterizing the cascade of early events if they are related to mineralization, odontogenesis or osteogenesis.

#### Effects of bead implantation (control pulps)

**Figure 1:**
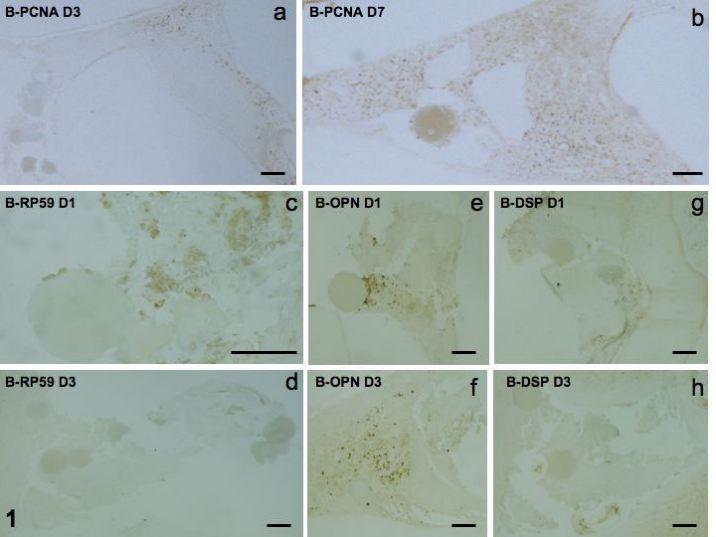
Implantation of beads alone. PCNA staining at day-3 and -7 (1a, b). RP59 at day -1 and -3 (1c, d). Immunostaining for osteopontin (OPN) at day -1 and -3 (1e, f), and for DSP for the same periods of time (1g, h).

Labeling for PCNA was increased between 1 and 7 days, indicating a steady cell growth during the repair process (Figs 1a, b). In contrast, the RP59 (presumed osteoblast/odontoblast differentiation marker) labeling level was weak on day 1 and was not increased at 3 and 7 days (Figs 1c, d). OPN, the marker for both cell inflammation and osteoblastic character was weak at day 1, moderately stronger at day 3 but not further enhanced by day 7 (Figs 1e, f). DSP labeling was negative at each time (Figs 1g, h).

#### Effects of implantation of beads soaked with A+4

**Figure 2:**
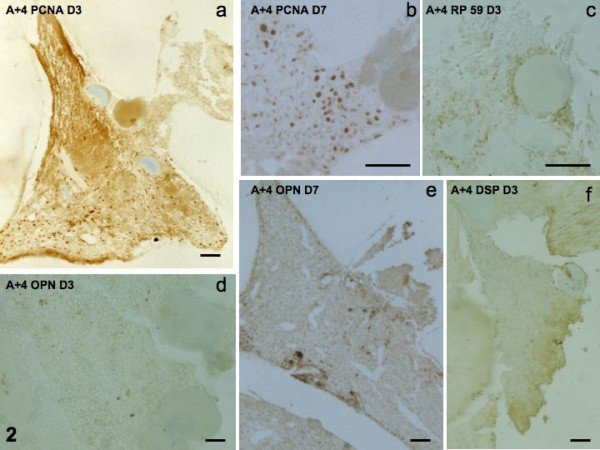
Implantation of agarose beads soaked in A+4. PCNA staining at day 3 and 7 (2a, b), RP59 at day 3 (2c), OPN at days 3 and 7 (2d, e). No staining is seen for DSP (2f).

No PCNA labeling was detectable at day 1. At day 3, labeling was strong, but had decreased sharply at day 7 (Figs 2a, b). Similarly, RP59 labeling was increased between days 1 and 3 but had declined at day 7 (Fig 2c). For the short periods of time studied here, OPN labeling was increasing between days 1–3, and reinforced at day 7 (Figs 2d,e), whereas DSP labeling was null all time periods (Fig 2f).

#### Effects of implantation of beads soaked with A-4

**Figure 3:**
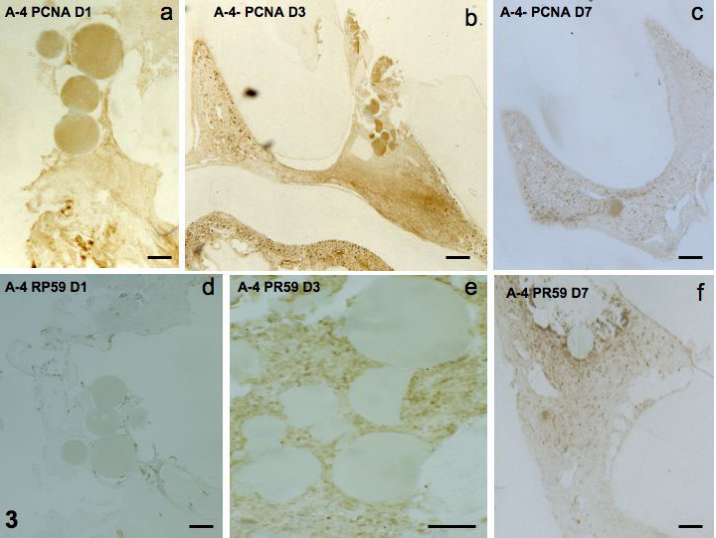
Implantation of A-4. PCNA at days 1, 3 and 7 (3a-c). RP59 staining at days 1, 3, and 7 (3d-f).

**Figure 4:**
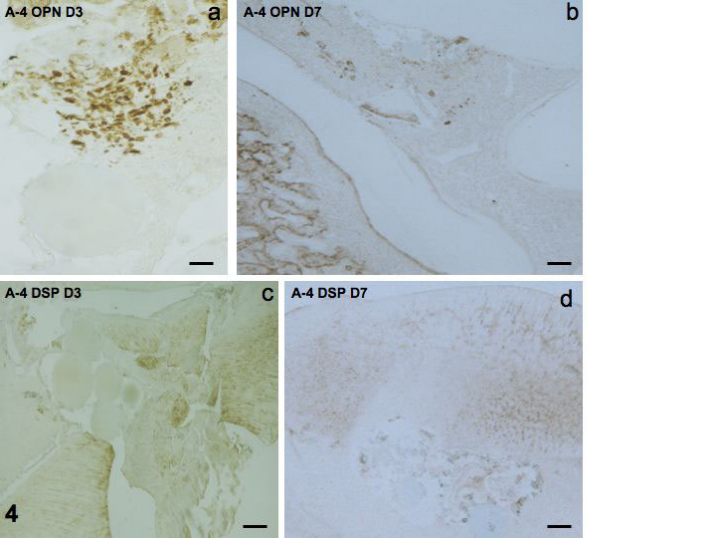
Immunostaining for OPN at days 3 and 7 (4a, b). DSP is negative at day 3 and a few cells are labeled at day 7 (4c, d).

PCNA labeling was weak at day 1, and enhanced at days 3 and 7 (Figs 3a–c). The same occurred for RP59 labeling (Figs 3d–f). OPN, negative at day 1, became positive at days 3 and 7 (Figs 4a, b). DSP labeling was undetectable at days 1–3, but had begun to display weak labeling of a few cells at day 7 (Figs 4c, d).

**Figure 5:**
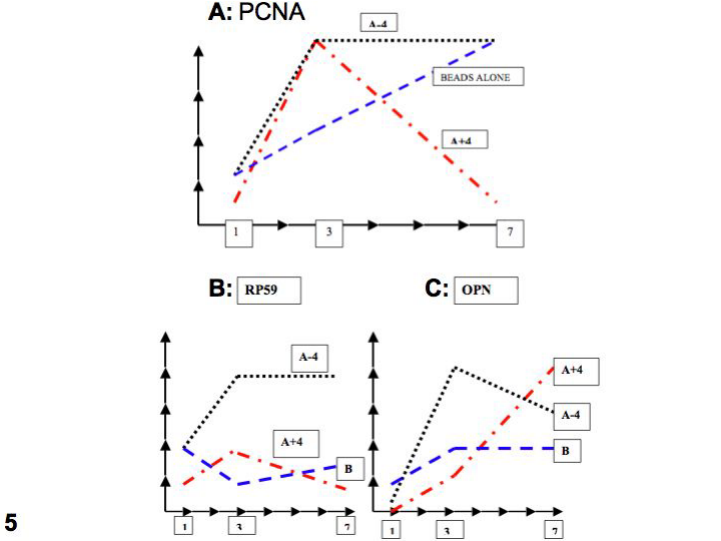
Figure 5A: Evaluation of PCNA immunostaining at 1, 3 and 7 days after implantation of beads alone or beads loaded with A+4 or A-4. RP59 (osteogenic differentiation) (5B) and osteopontin (OPN) (5C) immunolabeling for beads alone (B), beads loaded with A+4 and A-4, at 1, 3 and 7 days after implantation. No staining is scored 0; ± = 0.5; + = 1; between + and ++, depending on sections = 1.5; and ++ = 2.

Figures 5A–C reflect the difference in behavior of the four markers upon exposure of the pulp cells to the beads +/- amelogenin peptides. These data relative to PCNA indicate that while implantation of the beads alone does not impair the steady growth of the cells in the vicinity of the beads, the addition of A+4 or A-4 initially stimulates the cell growth rate, albeit in a differential fashion, with the A+4 effect dropping sharply after 7 days, while the A-4 effect is more long lasting (Fig 5A). Similar differential effects are seen for the osteogenic (RP59) and bone phenotypic (OPN) markers (Figs 5B, C). The effect of A+4 is markedly different from A-4 with regard to length of time for the development of the OPN response. The DSP appearance resulting from both A+4 and A-4 is not apparent until day 7, and then only to a limited extent in small groups of cells.

## Discussion

### Potential biological effects of agarose beads

The present results shed light on the short-term effects of agarose beads alone or beads soaked with A+4 or A-4 implanted in the exposed pulp. Each of the two bioactive molecules has its own specificity. Shortly after implantation of bioactive molecules in the dental pulp, it appears that some pulp cells differentiate along the osteoblast-like progenitor phenotype.

As agarose is a linear sulphated galactan, implantation of beads alone may have some effects on pulp repair. Antiviral and anticoagulant properties have been already reported [5,20]. They may interfere with the initial steps of pulp healing. A slight inflammatory reaction was detected, and pulp cell proliferation was initiated at day 3 and enhanced at day 7. However, none of the other markers provide any evidence for progenitor differentiation into osteoblast-like cells. Rodents have a tendency to self-repair and a limited spontaneous healing was observed after the surgery as reported previously [7]. However, healing after the surgery and bead implantation took longer periods of time, and was only partial even after 30 days, compared with the effects of two amelogenin molecules for the same period of time [33].

### Short-term effects of A +/- 4

How cells are committed still needs to be clarified, and probably an *in vitro *approach is more appropriate to further elucidate the mechanisms involved. Altogether, our data suggest that immediately after implantation of A +/- 4, the reparative process initiated by the two spliced amelogenins implicates three consecutive steps for the periods of time studied here.

Firstly, the committed cells proliferate, as shown by PCNA staining. These cells are located not only near the exposure site, but also in the central cusp at 3 days and 7 days. They migrate and after 7 days and at 15 days, labeled cells are seen surrounding the carrier beads. They are never located at the immediate surface, but they form the second row of the ring of cells located around the beads [33]. Dormant progenitors that are committed divide until the required number of cells is obtained to produce the ECM molecules necessary to form a reparative dentinal bridge or diffuse mineralization.

Secondly, the cascade of events pushes some cells toward an early differentiation of RP59-positive progenitor cells. As the RP59-positive cells are less numerous than the PCNA-positive cells, this suggests that all the cells are not transformed, but that some are selected according to unknown criteria.

As a third step, as shown by OPN immunostaining, some of the RP59-positive cells undergo the final differentiation toward the osteoblast-like lineage, despite implantation into the dental pulp which would in theory implicate their differentiation to odontoblast-like progenitors. The kinetics of the reaction supports the interpretation that in this case OPN, after the immediate inflammatory response from days 1 to 3, ceases to play its role as an inflammatory molecule, but rather characterizes osteoblast progenitors. Labeling for DSP is a late-occurring event, detect only after 7 days within a very few cells stimulated by A-4 alone. Either some osteoblast-like cells gradually acquire the odontoblast phenotype and display late terminal differentiation, or a second group of cells are recruited at a slower rate and they show a different phenotype. At the moment, *in vitro *data on clones of progenitors suggest that the cells are osteo/odontoblast progenitors [26], but when amelogenins are added to the culture medium, clones express DSP after 48 h both for A+4 and A-4, results divergent from the present *in vivo *data [19].

We have previously shown that 30 days after A+4 implantation, the formation of a reparative dentine bridge is observed, while A-4 stimulates the formation of a diffuse pulp mineralization occurring both in the crown and root parts of the pulp [33]. Therefore, the 14 amino acids that constitute the transcripts of exon 4 and make the difference between the M73, A+4 and M59, A-4, seem to constitute a specific domain regulating the speed and nature of the differing responses. In *in vitro *tissue culture experiments that examined the effects of the two bioactive molecules we saw that within 24 to 48 hours of their addition to the cells, upregulation of factors such as SOX9 and Runx2 was evident [40]. Although in vivo responses do take considerably longer times, the proteomics approach to examination of the early gene responses on a more global level will be required to understand the mode of action of the amelogenin peptides in detail.
